# Carob pod aqueous extract potentiates cisplatin efficacy and reduces toxicity in experimental hepatocellular carcinoma via mitochondrial and inflammatory pathway modulation

**DOI:** 10.1186/s40643-026-01029-0

**Published:** 2026-06-22

**Authors:** Wael Sobhy Darwish, Abada El Sayed Khadr, Maher Abd El Naby Kamel, Tamer A. Addissouky, Ahmed Zaki Ghareeb, Mabrouk A. Abd Eldaim, Mohand K. Razzaq, Ibrahim El Tantawy El Sayed, Hamed Mohamed Abdel-Bary, Doaa Ahmed Ghareeb

**Affiliations:** 1 Chemistry Department, Faculty of Science, Menoufia University, Shibin el Kom, Menoufia 32511 Egypt; 2 Department of Basic Science, College of Dentistry, Al Maaqal University, Al Basrah, 61003 Iraq; 3 Biochemistry Department, Medical Research Institute, Alexandria University, Alexandria, 21561 Egypt; 4 Research Projects unit, Pharos University in Alexandria, Alexandria, 21648 Egypt; 5 New Burg El-Arab Hospital, Ministry of Health, Alexandria, Egypt; 6 Center of Excellence for Drug Preclinical studies (CE-DPS), Pharmaceutical and Fermentation Industries Development Center (PFIDC), City of Scientific Research & Technological Applications (SRTA-City), New Borg El Arab, Egypt; 7 Egypt Center for Research and Regenerative Medicine (ECRRM), Cairo, Egypt; 8 Department of Biochemistry and Molecular Biology, Faculty of Veterinary Medicine, Menoufia University, Menoufia, 32511 Egypt; 9 Department of Biochemistry and Molecular Biology, Faculty of Veterinary 10. Medicine, Menoufia National University, Birket El Sabaa, Menoufia Egypt; 10 Department of Biochemistry, College of Medicine, University of Sumer, Thi-Qar, Iraq; 11 Bio-Screening and Preclinical Trial Lab, Biochemistry Department, Faculty of Science, Alexandria University, Alexandria, Egypt

**Keywords:** Hepatocellular carcinoma, Carob pod aqueous extract, Mitochondrial biogenesis, Oxidative stress, Combination therapy

## Abstract

**Background:**

Hepatocellular carcinoma (HCC) is a global health challenge with limited therapeutic options. The effectiveness of conventional medication like cisplatin is often compromised by their severe toxicity. This study investigated carob pod aqueous extract (CPAE), a polyphenol-rich natural product, as a potential adjunct therapy to enhance efficacy and mitigate cisplatin toxicity in a preclinical HCC animal model.

**Methods:**

A rat model of HCC was established using diethylnitrosamine (DEN) and carbon tetrachloride (CCl₄). Forty-two Wistar rats were divided into seven groups, receiving various treatments: control, CPAE, vehicle, HCC only, HCC+CPAE, HCC+cisplatin, and HCC+CPAE+cisplatin. Liver and kidney function, metabolic profiles, oxidative stress/antioxidant parameters, gene and protein expression (AMPK, PGC-1α, TFAM, SIRT1, iNOS, NF-κB, IκK, p53, SREBP-2), histopathology, and statistical analyses were performed.

**Results:**

HCC induction caused significant liver dysfunction, metabolic disturbances, oxidative stress, alongside dysregulation of AMPK/PGC-1α/TFAM and NF-κB/iNOS pathways. CPAE, alone or with cisplatin, markedly ameliorated these changes, improving liver and kidney function, restoring antioxidant status, reducing the tumor marker AFP, suppressing pro-inflammatory and oncogenic signaling, and enhancing histological architecture. Furthermore, Combination therapy demonstrated synergistic benefits, with CPAE reducing cisplatin-induced nephrotoxicity and enhancing its antitumor efficacy, primarily via modulation of mitochondrial biogenesis, redox balance, and inflammatory signaling.

**Conclusions:**

CPAE exhibits potent hepatoprotective and anti-HCC activity, especially when combined with cisplatin. This combination modulates mitochondrial and inflammatory pathways while mitigating cisplatin-induced toxicity. These finding position CPAE as a promising natural adjuvant for integrative HCC management. Further translational studies are warranted to validate these findings and explore clinical applicability.

**Graphical abstract:**

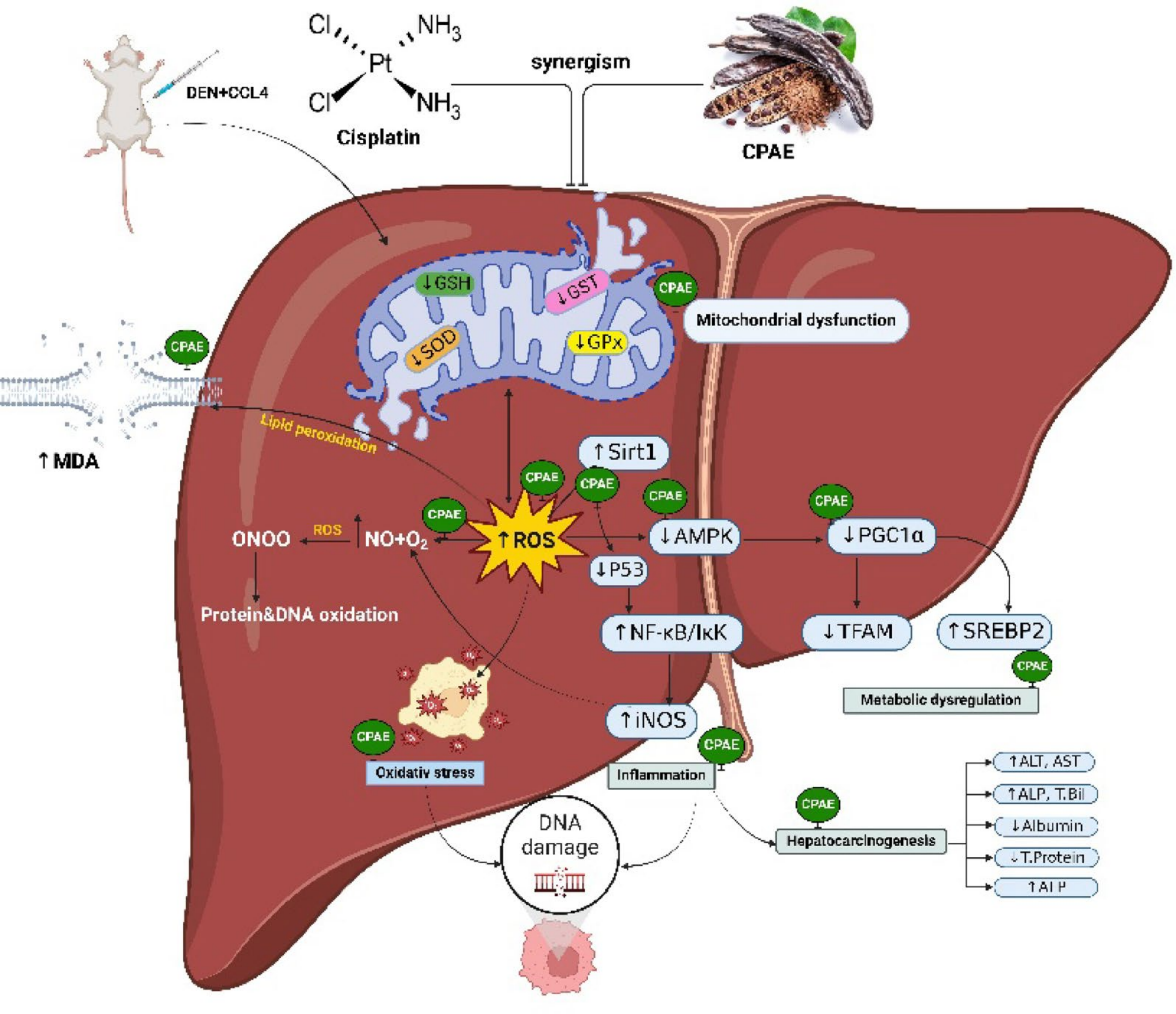

## Highlights

Carob extract significantly enhances the anticancer effects of cisplatin in a rat liver cancer model.

The combination therapy protects against liver and kidney damage while reducing chemotherapy toxicity.

Targeting mitochondrial and oxidative stress pathways offers a promising strategy for liver cancer treatment.

## Introduction

Primary liver cancer stands as the third most common cause of cancer-related mortality globally (Siegel et al. 2024). Hepatocellular carcinoma (HCC) is the most frequent form of primary liver cancer and develops in the context of chronic liver disease and cirrhosis with various etiologies. The major risk factors for the development of HCC include hepatitis B and C viruses, non-alcoholic steatohepatitis, obesity, excess alcohol consumption, and cigarette smoking (Zheng et al. 2025, Lee et al. 2021). In line with these risk factors and given the crucial role of mitochondria to hepatic function, growing evidence indicates that mitochondrial dysfunction as a likely central player in the pathological transition from hepatitis to HCC, through its association with the direct production of reactive oxygen species (ROS), apoptotic resistance, inflammation, and metabolic abnormalities. Furthermore, mitochondria play a critical role in cancer initiation or progression (Lee et al. 2021, Bian et al. 2021). Through these actions, AMPK switches off energy-consuming pathways while activating energy-producing pathways.

Among the key regulators of mitochondrial function and cellular energy homeostasis, AMP-activated protein kinase (AMPK) serves as a metabolic master switch in hepatocytes. It directly phosphorylates and regulates metabolic enzymes and nutrient transporters. AMPK indirectly stimulates the expression of nuclear genes involved in mitochondrial biogenesis and function., As a result, AMPK activates energy-producing pathways while switching off energy-consuming pathways (Hinchy et al. 2018, Rabinovitch et al. 2017). AMPK is considered a potential molecular target for cancer treatment, and its relevance in HCC has been demonstrated by its tumor-suppressive function. AMPK suppresses tumor growth by counteracting the Warburg effect and shifting cancer cell metabolism from glycolysis to oxidative phosphorylation. This metabolic reprogramming restores energy checkpoints and inhibits anabolic processes that drive proliferation (Rabinovitch et al. 2017, Sun et al. 2025). This is partly mediated through AMPK-PGC-1α, which stimulates mitochondrial biogenesis via the transcription factor TFAM [6, 65]. In addition to its antitumor role, the AMPK-PGC-1α axis mitigates chemotherapeutic toxicity. It confers cytoprotection against insults such as cisplatin by promoting mitochondrial biogenesis and enhancing TFAM expression, which collectively improve mitochondrial functions and redox homeostasis, thereby protecting against cisplatin-induced cytotoxicity in healthy tissues, particularly the kidneys [6, 43, 65].

Despite significant advances in the understanding the pathophysiology of HCC, therapeutic options remain limited. Chemotherapy represents one of the most frequent therapeutic regimens for advanced HCC. It is used to treat patients who are not candidates for surgical resection, local ablative therapy, or transarterial chemoembolization (Krupa et al. 2024). Cisplatin is one of the most commonly used chemotherapeutic drugs for the clinical treatment of hepatocarcinoma (Chen et al. 2021). Cisplatin is a non-specific cytotoxic agent that damage cells by forming platinum-DNA complexes, inhibiting DNA replication and transcription, and triggering apoptosis. However, its clinical use is limited due to severe dose-dependent toxicity, including nephrotoxicity, ototoxicity, neurotoxicity, and cardiotoxicity (Chen et al. 2021)(Dong et al. 2017). The nephrotoxicity is largely mediated by oxidative stress and mitochondria damage in the renal tubule (Elmorsy et al. 2024, Hong et al. 2025).

Therefore, the combination of cisplatin with antioxidants could alleviate or prevent these toxicities while enhancing its anticancer action (Elmorsy et al. 2024, Liu et al. 2025, Gao et al. 2023).

Natural products and their active constituents may affect the onset and development of liver cancer in a variety of ways, such as suppressing tumour cell development and metastasis, protecting against liver carcinogenic agents, immunomodulating, enhancing the effects of chemotherapeutic drugs, reducing oxidative stress, and chronic inflammation (Pan et al. 2020). Carob (*Ceratonia siliqua* L.) is an evergreen tree belonging to the plant family *Leguminosae*, cultivated or naturally grown in the Mediterranean region (Rtibi et al. 2017). It is a medicinal plant used in folk medicine to treat many ailments of the gastrointestinal tract, intestinal parasites, herpes lip sores, antitussives, menorrhagia, and warts. *Ceratonia siliqua* derives its medical value from its high content of secondary metabolites, which revealed that it possesses anti-inflammatory, anti-oxidant, anti-diabetic, antimicrobial, anti-diarrhea, anti-ulcer, anti-constipation, and anti-absorptive of glucose actions in the digestive system (Rasheed et al. 2019). Previously, we explored the contents of CPAE and its biological activities, and found that CPAE contained phenolics, flavonoids, alkaloids, amino acids, and carbohydrates. In addition, CPAE has antioxidant, anti-inflammatory, anti-viral, and anti-bacterial activities and has a hypoglycemic effect by inhibiting intestinal enzymes, maltase, lactase, sucrase, and amylase. Moreover, our results indicate that it is rich in gallic acid, which has potent antioxidant and anti-free radical scavenging activities and can protect biological cells, tissues, and organs from damage caused by oxidative stress (Darwish et al. 2021). Although carob pod aqueous extract (CPAE) possesses documented antioxidant and pleiotropic activities, its ability to enhance cisplatin efficacy while counteracting its nephrotoxicity has never been comprehensively evaluated in an in vivo HCC model. Furthermore, the capacity of CPAE to modulate redox homeostasis, mitochondrial biogenesis, and inflammatory signaling in the context of cisplatin remains largely unexplored. Thus, the present study was designed to evaluate the anti-carcinogenic potential of CPAE and cisplatin, alone and in combination, against chemically induced HCC in rats, with particular focus on elucidating their impact on oxidative stress, mitochondrial dysfunction, and inflammatory pathways, as well as the potential of CPAE to protect against cisplatin-induced nephrotoxicity.

## Materials and methods

### Animals and ethics statement

Forty-two male albino Wistar rats were used in this study (weight range: 150–170 g; age range: 2–3 months). The animals were procured from the Medical Research Institute’s Animal House at the University of Alexandria, Egypt. All rats had unrestricted access to food and water under a 12/12-hour light/dark cycle and constant environmental conditions before experimentation. The rats were observed for one week before the study. The experimental animals used in the study protocol were carried out following the ethical guidelines of the Medical Research Institute, University of Alexandria, Egypt (Approval No.: AU0122132423).

### Preparation of carob pod aqueous extract (CPAE)

The carob pods were gathered from the farms in New Borg El-Arab City, Alexandria, Egypt’s City of Scientific Research and Technological Applications (SRTA-City), washed with distilled water, and dried. Then, the CPAE was prepared as previously described by Darwish et al. (Darwish et al. 2021).

### Induction of hepatocellular carcinoma in rats

Hepatocellular carcinoma was induced as previously described by Uehara et al. (Uehara et al. 2014) with some modifications. The rats were injected intraperitoneally (IP) with DEN (100 mg/kg) diluted in 0.9 NaCl physiologic saline solution as a single dose, and then two weeks later, rats were injected (IP) with 0.25 mL/Kg CCl_4_ diluted in olive oil (1:3) twice weekly for 32 weeks. Histopathological examination and determination of the level of alpha-fetoprotein (AFP) were employed to confirm the presence of HCC.

## Experimental design

The animals were divided into seven groups with the ‘n’ number for each group (*n* = 6 per group, total *n* = 42) as illustrated in Table 1.

- Group Ⅰ: normal control rats were fed with a normal basal diet and were neither treated nor injected for 36 weeks.
- Group Ⅱ: normal rats were fed a normal basal diet for 32 weeks before being given carob pod aqueous extract (CPAE) 200 mg/Kg BW dissolved in distilled water daily via oral gavage tube for an additional 4 weeks, totaling 36 weeks (Elhalim et al. 2017).
- Group Ⅲ: Vehicle normal rats were injected IP with a single dose of normal saline and after 2 weeks were injected IP with 0.25 mg/kg BW of olive oil twice a week for 32 weeks. Besides a normal basal diet for 36 weeks.
- Group Ⅳ: HCC-induced rats that did not receive any treatment.
- Group Ⅴ: HCC rats treated orally with CPAE at a dose of 200 mg/kg BW daily for 4 weeks.
- Group Ⅵ: HCC+Cisplatin: HCC rats were treated with cisplatin at a dose of 0.5 mg/kg BW, administrated IP twice weekly for 4 consecutive weeks.
- Group Ⅶ: HCC+CPAE+Cisplatin: HCC rats treated with a combination of 0.5 mg/kg BW cisplatin administered IP twice per week and 200 mg/kg BW CPAE daily for 4 weeks.

**Table 1:** Experimental design and treatment groups

Group	Group description	HCC induction	Treatment intervention	Treatment duration	Total study period
I	Normal control	Normal (No HCC)	No treatment	None	36 Weeks
II	Normal + CPAE	Normal (No HCC)	Treated with CPAE 200 mg/Kg BW dissolved in distilled water daily via oral gavage tube [18]	4 weeks	36 weeks
III	Vehicle control	Normal (No HCC)	Rats were injected IP with a single dose of normal saline and after 2 weeks were injected IP with 0.25 mg/kg BW of olive oil twice a week for 32 weeks	None	36 weeks
IV	HCC control	Hepatocellular carcinoma (HCC)-induced	No treatment	None	36 weeks
V	HCC + CPAE	HCC-Induced	HCC rats treated orally with CPAE at a dose of 200 mg/kg BW daily	4 weeks	36 weeks
VI	HCC + cisplatin	HCC-Induced	HCC rats were treated with cisplatin at a dose of 0.5 mg/kg BW, administrated IP twice weekly	4 weeks	36 weeks
VII	HCC + CPAE + cisplatin	HCC-induced	HCC rats treated with a combination of 0.5 mg/kg BW cisplatin administered IP twice per week and 200 mg/kg BW CPAE daily	4 weeks	36 weeks

By the end of the therapeutic time, overnight fasting rats were euthanized by deep isoflurane inhalation following the ethical guidelines approved by the institutional Animal Care and Use Committee. Blood samples were collected and then centrifuged for ten minutes at 1000xg at room temperature to obtain serum for the assessment of biochemical parameters. The liver tissues of the animals were quickly removed and washed with ice-cold saline and blotted dry. The left lobe of the liver was fixed in 10% formalin for histological investigations. While the right lobe was stored at −80 °C for assessment of biochemical and molecular analysis.

### Biochemical assays

Liver serum parameters aspartate aminotransferase (AST), alanine aminotransferase (ALT), and alkaline phosphatase (ALP) were measured using kinetic methods with commercially available kit (BIOLABO S.A.S., FRANCE). Enzymatic coulometric assays were utilized to estimate total protein, bilirubin, albumin, total cholesterol (TC), triglycerides (TGs), and fasting blood sugar (FBS) using the same kit provider. Absorbance reading was performed using autoanalyzer BS-240 (China). Kidney function test; Blood urea was determined using enzymatic urease-based colorimetric assay, in which urea hydrolyzed to ammonia, then followed by formation green chromogenic indophenol complex, and serum creatinine level was estimated according to the Jaffé kinetic method using commercially available kit (Vitro, Egypt) with the same instrument. AFP levels in rat serum quantified by enzyme-linked immune-sorbent assay (ELISA) using a Biospes kit (China) cat NO (BYEK2963). Serum samples were dilute 1:5 in serum buffer. Optical density was measured at 450 nm in a microplate ELISA reader. The analytical sensitivity range of the ELISA kit was 0.1to 50 ng/mL. All assays were conducted in triplicate and according to the procedure described by the manufacturer’s instructions.

### Oxidative stress markers

#### Determination of hepatic malondialdehyde

Hepatic oxidative stress was evaluated by quantification of malondialdehyde (MDA) as a marker of lipid peroxidation using a method described by Tappel and Zalkin (Tappel and Zalkin 1959), . Briefly, 0.5 mL of the liver homogenate was added to 1 mL TCA and centrifuged at 1000x g for 10 min at 4 °C. To 1 mL of supernatant, 0.5 mL of TBA (0.7%) was added and heated in a boiling water bath for 45 min forming a pink MDA-TBA adduct. The blank was prepared using 0.5 mL of distilled water instead of liver homogenate, and then the obtained color was estimated spectrophotometrically at 532 nm against the blank. The MDA levels were expressed in nmol/mg protein.

#### Determination of hepatic nitric oxide (NO)

The nitric oxide (NO) level in liver homogenate was determined using the method described by Bryan and Grisham, Briefly, 100 µL of liver homogenate (test), standard nitrite solution (standard), or distilled water (blank) was added to one mL of sulphanilamide. The mixtures were incubated for 5 min at room temperature. Subsequently, 100 µL of N-(1-naphthyl) ethylenediamine was added to all test tubes and incubated for 20 min at room temperature. The absorbance of the test and standard were measured at 540 nm against blank (Bryan and Grisham 2007).

#### Determination of hepatic xanthine oxidase (XO)

Hepatic tissue xanthine oxidase (XO) activity was assessed in liver homogenates according to the method of Litwack et al. (Litwack et al. 1953). Briefly, 0.5 mL of liver homogenate was incubated at 37 °C for 20 min. Following this, 30 µL of buffer and 60 µL xanthine stock solution (test) or 60 µL distilled water (control) was added and the mixture was incubated for 40 min at 37 °C. After incubation, 0.1 mL of this mixture was mixed with 0.1 mL sodium tungstate, 0.5 mL distilled water, and 0.1 mL sulfuric acid, and the volume was adjusted to 1 mL with distilled water and incubated for 1 h at 37 °C. The tubes were centrifuged, and 0.15 mL of supernatant was mixed with 0.75 mL of distilled water, 0.3 mL of folin’s reagent, and 1.5 mL of sodium carbonate. A blank was prepared by replacing the reaction mixture with distilled water. while the standard was prepared using the same procedure as the blank method except that 0.1 mL of standard was added instead of 0.1 mL of water in the first step. The absorbance of the samples and standard was measured at 660 nm against the reagent blank.

### Hepatic antioxidant parameters

#### Determination of hepatic glutathione (GSH)

The hepatic glutathione (GSH) levels were assessed using the method of Jollow et al. Briefly, 0.1mL of liver homogenate (test), dH_2_O (blank), and known GSH (standard) were mixed with 0.1 mL of sulfosalicylic acid. The samples were incubated at 4 °C for at least one hour and then centrifuged at 1000x g rpm for 10 min at 4 °C. Then 0.1 mL of supernatant was mixed with 2.7 mL phosphate buffer and 0.2 mL DTNB and incubated for 5 min. The developed yellow color was immediately measured at 412 nm (Jollow et al. 1974).

#### Determination of hepatic glutathione peroxidase (GPx)

Glutathione peroxidase (GPx) activity in hepatic tissue was evaluated according to the method of Pierce and Tappel (Pierce and Tappel 1978). Briefly, the test sample was prepared by adding 50 µL of the diluted homogenate supernatant to a mixture of 100 µL GSH, 100 µL cumene hydroperoxide, and 750 µL Tris-HCl buffer, pH 7.6. The control was prepared as a test without adding cumene. Both test and control mixtures were incubated at 37 °C for 10 min. One milliliter of TCA was added to both tubes, and 100 µL of cumene H_2_O_2_ was added to the control tube. Then, both tubes were centrifuged at 3000 × g for 20 min. Subsequently, 1 mL of supernatant from each tube was mixed with 2 mL of Tris-HCl, pH 8.9, and 100 µL DTNB. The mixture was incubated for 5 min at room temperature, and the absorbance was measured at 412 nm against a distilled water blank.

#### Determination of glutathione -S- transferase (GST)

Hepatic glutathione-S-transferase (GST) activity was determined spectrophotometrically according to the method described by Habig et al. (Habig et al. 1974). The assay was performed by incubating 25 µL of liver homogenate with a mixture containing 100 µL GSH, 10 µL of p-nitrobenzyl chloride, and 1.365 mL of phosphate buffer, pH 6.5, for 20 min at room temperature. The absorbance of the samples was measured against air at 310 nm. spectrophotometrically based on the inhibition of pyrogallol auto-oxidation.

#### Determination of hepatic superoxide dismutase (SOD)

Determination of hepatic superoxide dismutase (SOD)-specific activity was evaluated spectrophotometrically based on the inhibition of pyrogallol auto-oxidation according to the method described by Marklund & Marklund (Marklund and Marklund 1974). In one cm quartz cuvette, 20 µL of liver homogenate (test) or buffer (reference) and 10 µL pyrogallol were added to 1mL buffer solution. The absorbance of the test (At) or reference (Ar) was read at 420 nm against air after 30 s and 90 s.

#### Determination of hepatic ATPase activity

Total ATPase activity was determined by measuring inorganic phosphate released from ATP hydrolysis, using a standard phosphomolybdate assay (Candeias et al. 2009). Briefly, the liberated Pi react with ammonium molybdate in the presence of sulfuric acid to form a chromogenic complex measured at 430 nm. The biochemical parameters MDA, NO, GST, SOD, GSH, and ATPase were standardized to the total protein content of the liver homogenates the concentration of protein was determined using the method of Lowery et al. (Wu et al. 2025) with bovine serum albumin (BSA, 1 mg/mL) as a reference standard (Candeias et al. 2009).

##### Assessment of nuclear factor kappa B (NF-κB), IκK, phosphorylated P53, and sterol regulatory element binding protein-2 (SREBP-2)

The levels of total NF-κB, IKK, pP53 and SREBP2 were quantified individually in liver tissue homogenate supernatant using sandwich ELISA kits (MyBioSource, San Dieego, CA, USA) following the manufacture protocols. Liver tissues were homogenized in ice cold in 1x PBS (10 mM Na_2_HPO_4_, 2 mM KH_2_PO_4_, 2.7 mM KCl, 137 mM NaCl, pH 7.4) supplemented with 1x phosphatase and1x protease inhibitors. The homogenates were the centrifuged for 15 min at 12.000xg at 4 °C, and the obtained supernatant was collected for ELISA analysis. The total protein concentration of each sample was quantified using Bradford assay. All ELISA assays were performed in duplicate and expressed as ng/mL.

##### Gene expression analysis using RT-PCR

Quantitative analysis of AMPK, PGC-1α, sirtuin-1 (SIRT1), TFAM, and inducible nitric oxide synthase (iNOS) gene expression in liver tissues was performed using quantitative real-time reverse transcriptase-polymerase chain reaction (qRT-PCR). First, the miRNeasy Mini Kit (Qiagen, Germany) was used for the extraction of the total RNA from the tissues in accordance with the manufacturer’s instructions. Next, the reverse transcriptase enzyme was used to create complementary DNA (cDNA) from the extracted RNA using the QuantiTect Reverse Transcription Kit (Qiagen, Germany) according to the manufacturer’s instructions, and then cDNA was amplified and detected using particular primers Table 2 by real-time PCR. The quantitative PCR assay was performed by Rotor-Gene SYBR Green PCR Kit (Qiagen^®^, Germany), and the data was collected using Rotor-Gene Q-Pure Detection version 2.1.0 (build 9) (Qiagen, Valencia, CA, USA). The relative expression of AMPK, PGC-1α, TFAM, SIRT1, and iNOS was quantified relative to the expression of the reference gene 18s rRNA in the same sample by calculating and normalizing the Ct values of target genes to those of 18s rRNA using the ΔΔCt method (Livak and Schmittgen 2001).

**Table 2:** Forward (F) and reverse primer (R) sequence of AMPK, PGC-1α, TFAM, SIRT1, iNOS, and 18s rRNA (Reference gene)

Genes	Accession no.	Primer sequence	
AMPK	NM_023991.1	F R	GTGGTGTTATCCTGTATGCCCTTCT CTGTTTAAACCATTCATGCTCTCGT
PGC-1α	NM_031347.1	F R	GTGCAGCCAAGACTCTGTATGG GTCCAGGTCATTCACATCAAGTTC
TFAM	NM_031326.2	F R	CCCTGGAAGCTTTCAGATACG AATTGCAGCCATGTGGAGG
SIRT1	NM_001372090.1	F R	TGTTTCCTGTGGGATACCTGA TGAAGAATGGTCTTGGGTCTTT
iNOS	NM_012611.3	F R	CACCACCCTCCTTGTTCAAC CAATCCACAACTCGCTCCAA
18 S rRNA	NR_046237.2	F R	GTAACCCGTTGAACCCCATT CAAGCTTATGACCCGCACTT

##### Western blot of nuclear factor kappa B (NF-κB), IκK, phosphorylated P53, and sterol regulatory element binding protein-2 (SREBP-2)

Briefly, 50 µg protein lysates from each sample were mixed with 2X loading buffer (130 mM Tris-HCl, pH 8.0, 30% (v/v) Glycerol, 4.6% (w/v) SDS, 0.02% Bromophenol Blue, and 2% DTT). Samples were boiled for 5 min, then cooled at 4 °C. Samples were separated on 12% SDS-PAGE mini-gel (1.6 mL H2O, 1.5 M Tris pH 8.8, 1.3 mL of 30% Acrylamide acrylamide/bisacrylamide (29:1 mix in 100 mL), 2.0 mL 10% SDS, 10% APS, TEMED 2 mL) and run at 120 V. A nitrocellulose membrane was used to transfer proteins at 22 V overnight at 4 °C. The membrane was washed three times with TBST (50 mM Tris, pH 7.5, 150 mM NaCl, 0.05% Tween-20) and then incubated in blocking buffer (TBST containing 5% nonfat dry milk) for 1 h at RT. Primary antibodies diluted (1:1000 in Tris-buffered saline containing Tween 20 (TBST) and 5% bovine serum albumin (BSA)) targeting (NF-κB p65 (Cell signaling, cat No; C22B4), IκKү (Cell Signaling, cat No; #2685), P53 (Cell Signaling, cat No; (7F5) Ab 2527) phosphorylated P53 (Sigma-Aldrich, cat No; PSER15), and SREBP-2 (Abcam, cat No; ab30682)) were incubated over night at 4 °C. After three washes with TBST, the membrane was incubated with a secondary antibody for 1 h at RT. Membranes were washed three times with TBST again. The bands were detected by an alkaline phosphatase solution (Burnette 1981). Each group was represented as one sample for confirmation of the ELISA results.

### Histopathological investigations

The formalin-fixed liver specimens were processed in an automated tissue processor. The processing consisted of two initial steps of fixation and dehydration. Fixation comprises tissue immersion in 10% buffered formalin for 48 hours, followed by fixation removal in distilled water for 30 minutes. Then, dehydration was carried out by running the tissues through a series of alcohol concentrations (70%, 90%, and 100%). The tissue was first exposed to 70% alcohol for 120 minutes, followed by 90% alcohol for 90 minutes, and then two cycles of absolute alcohol, each lasting one hour. The tissues were then cleared with a graded concentration of xylene. It consisted of immersion of the tissue for an hour in a mixture of 50% alcohol and 50% xylene, followed by immersion in pure xylene for 1.5 hours. Samples were then impregnated using molten paraffin wax at 56–58 °C and embedded to form tissue blocks. Paraffin sections with 4 µm thickness were cut, and paraffin was removed with xylene. The specimen is rehydrated through descending alcohols (100%, 90%, 70%), and slides were stained using hematoxylin for 10 minutes to stain nuclei with blue color and eosin for 10 seconds to stain cytoplasm with red color. Stained sections were dehydrated using ascending grades of alcohol for 10 minutes. All traces of alcohol were removed using a clearing agent (xylene) for 15 minutes. adding a synthetic mounting medium, DPX, to a cover slip and then placing it on the top of the sections. Then the slides were examined under a light microscope.”.

### Synergy determination

The nature of the pharmacological interactions between CPAE and cisplatin was quantitatively assessed using the combination index (CI) methodology described by Li Li [REF 1].

This model quantifies drug interactions as synergistic, additive, or antagonistic. The predictive value for an additive effect was derived from the following formula:

$$ \begin{gathered} {\mathrm{The}}\;{\mathrm{predicted}}\;{\mathrm{value}} \hfill \\ \quad = \left( {\frac{{{\mathrm{Observed}}\;{\mathrm{value}}\;{\mathrm{of}}\;{\mathrm{CPAE}}}}{{{\mathrm{Control}}\;{\mathrm{value}}}}} \right. \hfill \\ \left. {\quad \quad + \frac{{{\mathrm{Observed}}\;{\mathrm{value}}\;{\mathrm{of}}\;{\mathrm{Cisplatin}}}}{{{\mathrm{Control}}\;{\mathrm{value}}}}} \right) \hfill \\ \quad \quad *{\mathrm{Control}}\;{\mathrm{Value}} \hfill \\ \end{gathered} $$

The combination index (CI) was then determined as (Lowry et al. 1951)

$$\mathrm{C}\mathrm{I}=\frac{\mathrm{O}\mathrm{b}\mathrm{s}\mathrm{e}\mathrm{r}\mathrm{v}\mathrm{e}\mathrm{d}\mathrm{v}\mathrm{a}\mathrm{l}\mathrm{u}\mathrm{e}}{\mathrm{P}\mathrm{r}\mathrm{e}\mathrm{d}\mathrm{i}\mathrm{c}\mathrm{t}\mathrm{e}\mathrm{d}\mathrm{v}\mathrm{a}\mathrm{l}\mathrm{u}\mathrm{e}\mathrm{o}\mathrm{f}\mathrm{c}\mathrm{o}\mathrm{m}\mathrm{b}\mathrm{i}\mathrm{n}\mathrm{a}\mathrm{t}\mathrm{i}\mathrm{o}\mathrm{n}}$$

According to this model, interactions are defined as follow Antagonistic ^>^1, synergestic^<^1, additive = 1 (Shaban et al. 2020).

### Statistical analysis

The statistical evaluation of the data was carried out using One-way ANOVA and Tukey’s post hoc multiple range tests via GraphPad Prism 5 (GraphPad, San Diego, CA, USA). *p*-value < 0.05 was considered statistically significant.

## Results

### Impact of CPAE and/or cisplatin on liver function and tumor markers

As shown in Table 3, the healthy rats supplemented with CPAE, or the vehicle group showed no significant changes in all liver function tests compared to the control group. In contrast, the HCC rats showed significant elevations in the serum activities of ALT, AST, and ALP and the level of bilirubin compared to the control group. These changes were accompanied by significant reductions in the levels of total protein and albumin. Moreover, serum AFP levels were significantly higher in HCC groups than the control groups. The treatment of HCC rats with CPAE, cisplatin, or their combination significantly ameliorated all the liver function tests except serum albumin level, which was significantly ameliorated in the HCC rats treated with CPAE only. The treatment with CPAE showed the best effect in ALT, ALP, Albumin, and bilirubin. According to the calculated CI, the combined treatment with CPAE/Cisplatin exhibited a synergistic impact on the level of AST, T-Protein, Albumin, and AFP Table 5).

**Table 3:** Biochemical liver parameters in different studied groups

Control	ALT(U/L)	AST(U/L)	AlP(U/L)	Albumin (g/dL)	T-protein (g/dL)	Bilirubin (mg/dL)	AFP (ng/mL)
	78.5^d^ ± 9.29	161.7^b^ ± 13.8	232.5^c^ ± 8.73	4.73^a^±0.38	9.0^a^ ± 0.11	0.19^d^±0.11	1.92^d^±0.20
CPAE	74.2^d^ ± 5.43	158.2^b^ ± 10.7	213.7^c^ ± 7.54	4.85^a^±0.17	9.12^a^±0.17	0.20^d^±0.07	2.0^d^ ± 0.08
Vehicle	76^d^±5.16	159.5^b^ ± 9.57	239.5^c^ ± 8.5	4.49^ac^±0.20	9.0^a^ ± 0.18	0.19^d^±0.009	1.95 ^d^±0.17
HCC	162.7^a^ ± 12.6	223.5^a^ ± 16.9	326.7^a^ ± 19.3	3.30^b^±0.08	7.57^c^±0.09	0.91^a^±0.88	9.80^a^±0.29
HCC+CPAE	92.25^cd^±7.8	162^b^±15.8	229^c^±16.2	3.93^cd^±0.11	8.5^b^ ± 0.16	0.43^bc^±0.17	6.10^b^±0.16
HCC+cisplatin	128^b^±13.1	166^b^±12.6	286.2^b^ ± 9.1	3.47^bd^±0.22	8.4^b^ ± 0.14	0.59^b^±0.09	6.07^b^±0.35
HCC+Combination	115^bc^±9.1	159.2^b^ ± 11.5	232.5^c^ ± 19.7	3.82^d^±0.12	8.6^ab^ ±0.15	0.67^b^±0.14	4.86^c^±0.30

Data were expressed as mean ± SD, *n* = 6. Mean values in the same column with common superscript letters (a, b,c, d) are not significant while mean values with different superscript letters are significantly different using the one-way ANOVA Test followed by the Tukey post hoc test at *p* > 0.05.

### Impact of CPAE and/or cisplatin on serum kidney function and metabolic profile

1. Kidney function results

The HCC rats showed significant elevations in the serum levels of creatinine and blood urea compared to the control, CPAE, and vehicle groups. The treatment of HCC rats with CPAE significantly ameliorated kidney function tests, while HCC rats treated with cisplatin alone or in combination with CPAE did not significantly reduce creatinine or urea levels, which remained elevated compared to control, CPAE, and vehicle groups. The best effects were observed in the HCC rats treated with CPAE alone Table 4.

2. Metabolic and lipid profile results

Regarding metabolic parameters, the supplementation of healthy rats with CPAE or vehicle didn’t show any significant changes in FBS and TG compared to control rats. On the other hand, the healthy rats supplemented with CPAE showed a significant decline in cholesterol compared to the control and vehicle. The HCC untreated rats showed a significant elevation in fasting glucose compared to the control, CPAE, and vehicle groups. Whereas cholesterol and TG levels were significantly higher only when compared to CPAE-treated healthy rats, but not to control or vehicle groups. The treatment of HCC rats with CPAE, cisplatin, or their combination significantly ameliorated fasting blood glucose and lipid parameters, the best effects being observed in the HCC rats treated with CPAE alone Table 4.

**Table 4:** Kidney function and metabolic parameters in different studied groups

Control	Urea (mg/dL)	Creatinine (mg/dL)	TG (mg/dL)	Cholesterol (mg/dL)	FBS (mg/dL)
	40^d^±5.09	0.49^c^±0.068	129.75^b^±7.45	120^a^±6.97	79.25^b^±6.99
CPAE	42.5^d^ ±6.06	0.45^c^±0.08	116.5^b^ ± 1.29	86.5^b^ ± 8.69	72.5^b^ ± 5.44
Vehicle	44^d^±4.69	0.53^c^±0.16	133.25^b^±4.78	113^a^±6.27	77.57^b^.04
HCC	58.5^ab^±2.9	1.12^a^±0.17	154.7^a^ ± 11.7	125.2^a^ ± 6.94	111^a^±9.20
HCC+CPAE	49.2^cd^±5.18	0.77^bc^±0.02	126^b^±5.35	96.5^b^ ± 9.71	82.25^b^±13.3
HCC+cisplatin	69.2^a^ ± 7.46	1.05^a^±0.14	115.2^b^ ± 16.1	116.5^a^ ± 9.98	77.25^b^±2.62
HCC+Combination	55^bc^±4.24	0.93^a^±0.04	115^b^±8.6	122.5^a^ ± 3.1	82^b^±1.82

Data were expressed as mean ± SD, *n* = 6. Mean values in the same column with common superscript letters (a, b,c, d) are not significant while mean values with different superscript letters are significantly different using the one-way ANOVA Test followed by the Tukey post hoc test at *p* > 0.05.

### Impact of CPAE and/or cisplatin on antioxidant and oxidative stress parameters

1. Antioxidant markers

Data presented in Table 5 showed significant depletion in the activity of SOD, GSH, GST, GPX, and ATPase in HCC rats compared to the control, CPAE, and vehicle. The treatment of HCC rats with CPAE, cisplatin, or their combination significantly ameliorated all the antioxidant parameters, while SOD, GSH, and ATPase in the cisplatin group were still significantly lower than those of the control, vehicle, and CPAE groups. Combination therapy showed the best effect in GSH, GST, and GPX.

2. Oxidative stress markers

On the other hand, the HCC rats showed significant elevations in the levels of NO, MDA, and the activity of XO compared to the control, CPAE, and vehicle groups. While the treatment of HCC rats with CPAE, cisplatin, or their combination ameliorated all the oxidative stress parameters. Interestingly, CPAE treatment demonstrated superior effects, and when combined with cisplatin, it enhances cisplatin potential, exerting a synergistic impact on most oxidative stress and antioxidant parameters. This was confirmed by the calculated CI values (CI < 1), as shown in Table 5.

**Table 5:** Antioxidants and oxidative stress parameters in different studied groups

Control	GSH (mg/mg.*P*)	GST (U/mg.*P*)	GPX (U/mg.*P*)	SOD (U/mg.*P*)	ATPase (nmol/mg.*P*)	MDA (nmol/mg.*P*)	NO (nmol/mg.*P*)	XO (U/mg.*P*)
	1.92^a^±0.18	6.82^a^±0.66	3.43^a^±0.45	1.90^a^±0.17	1.49^a^±0.08	8.55^d^±0.57	211.5^e^ ± 15.2	336.0^d^ ± 26.4
CPAE	2.27^a^±0.47	6.69^a^±0.22	3.27^a^±0.34	1.95^a^±0.17	1.58^a^±0.13	7.75^d^±0.35	203^e^±11.5	320.2^d^ ± 22.1
Vehicle	2.0^a^ ± 0.27	6.44^a^±0.22	3.15^a^±0.3	1.93^a^±0.17	1.47^a^±0.24	7.83^d^±0.80	210.5^e^ ± 18.5	341.5^d^ ± 21.9
HCC	1.03^d^±0.11	4.64^b^±0.44	1.15^d^±0.04	1.02^c^±0.07	0.62^d^±0.09	20.83^a^±1.13	913.4^a^ ± 32.4	602.5^a^ ± 31.5
HCC+CPAE	1.74^b^±0.08	6.32^a^±0.09	2.37^c^±0.27	1.72^b^±0.18	1.18^b^±0.19	10.82^c^±0.80	336.0^d^±31	372.7^cd^±21.6
HCC+cisplatin	1.26^c^±0.10	5.98^a^±0.48	2.12^c^±0.47	1.38^b^±0.12	0.85^c^±0.10	13.03^b^±0.76	585.7^b^ ± 25.5	452.2^b^ ± 32.1
HCC+Combination	1.95^a^±0.16	6.33^a^±0.28	2.66^bc^±0.05	2.0^a^ ± 0.15	1.06^bc^±0.104	11.95^b^±0.22	498.2^c^ ± 19.9	389.5^c^ ± 11.01

Data were expressed as mean ± SD, *n* = 6. Mean values in the same column with common superscript letters (a, b,c, d) are not significant while mean values with different superscript letters are significantly different using the one-way ANOVA Test followed by the Tukey post hoc test at *p* > 0.05.

### Impact of CPAE and/or cisplatin on hepatic gene expression parameters of AMPK, PGC-1α, TFAM, SIRT1, and iNOS

**Fig. 1:**
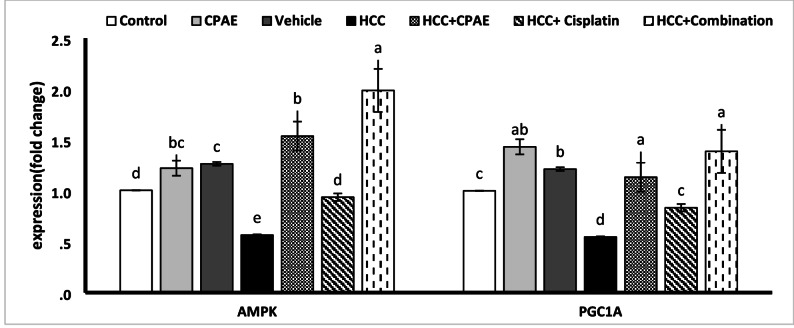
Hepatic gene expression of AMPK and PGC-1α in different studied groups. Data were expressed as mean ± SD, *n* = 6. Mean values in the same column with common superscript letters (a, b,c, d) are not significant while mean values with different superscript letters are significantly different using the one-way ANOVA Test followed by the Tukey post hoc test at *p* > 0.05

**Fig. 2:**
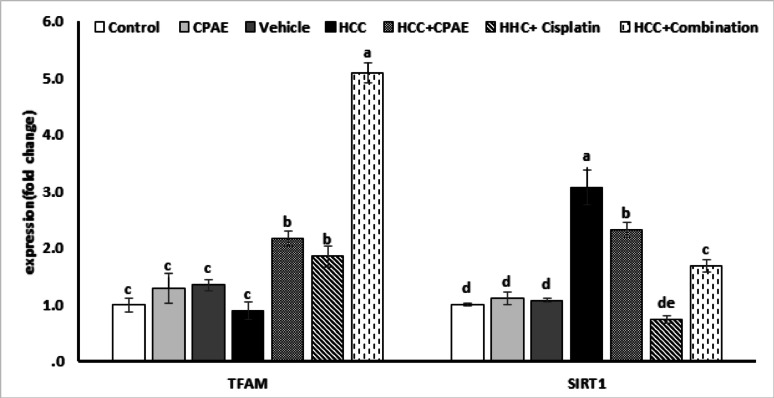
Hepatic gene expression of TFAM and SIRT1 in different studied groups. Data were expressed as mean ± SD, *n* = 6. Mean values in the same column with common superscript letters (a, b, c, d, e) are not significant while mean values with different superscript letters are significantly different using the one-way ANOVA Test followed by the Tukey post hoc test at *p* > 0.05

**Fig. 3:**
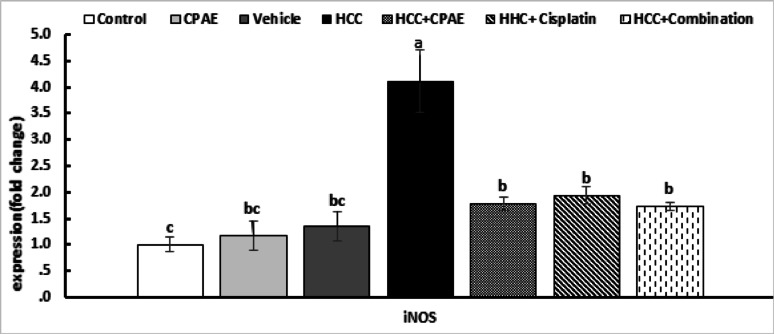
Hepatic gene expression of iNOS in different studied groups. Data were expressed as mean ± SD, *n* = 6. Mean values in the same column with common superscript letters (a, b, c, d) are not significant while mean values with different superscript letters are significantly different using the one-way ANOVA Test followed by the Tukey post hoc test at *p* > 0.05

 Figures (1, 2 and 3), show the hepatic mRNA expression levels of AMPK, PGC-1α, TFAM, SIRT1, and iNOS. Healthy rats supplemented with CPAE or the vehicle didn’t show any significant changes in all hepatic gene expression. On the contrary, the HCC rats showed significant up-regulation of SIRT1 and iNOS, where iNOS increased 4 folds and SIRT1 increased 3 folds compared to the control group. Conversely, the HCC rats showed significant downregulation of AMPK and PGC-1α expression compared to the control, CPAE, and vehicle groups. TFAM expression didn’t significantly differ from control, CPAE, and vehicle. The treatment of HCC rats with CPAE, cisplatin, or their combination significantly corrected the dysregulation of all hepatic gene expression compared to control, CPAE, and vehicle rats. SIRT1 expression remained significantly higher than control in HCC rats treated with CPAE alone. Combination therapy showed the most pronounced corrective effect overall. The CI for CPAE/Cisplatin was less than one which indicates its synergistic impact (Table 5).

### Impact of CPAE and/or cisplatin on hepatic protein parameters of NF-κB, IκK, pP53, and SREBP-2

**Fig. 4:**
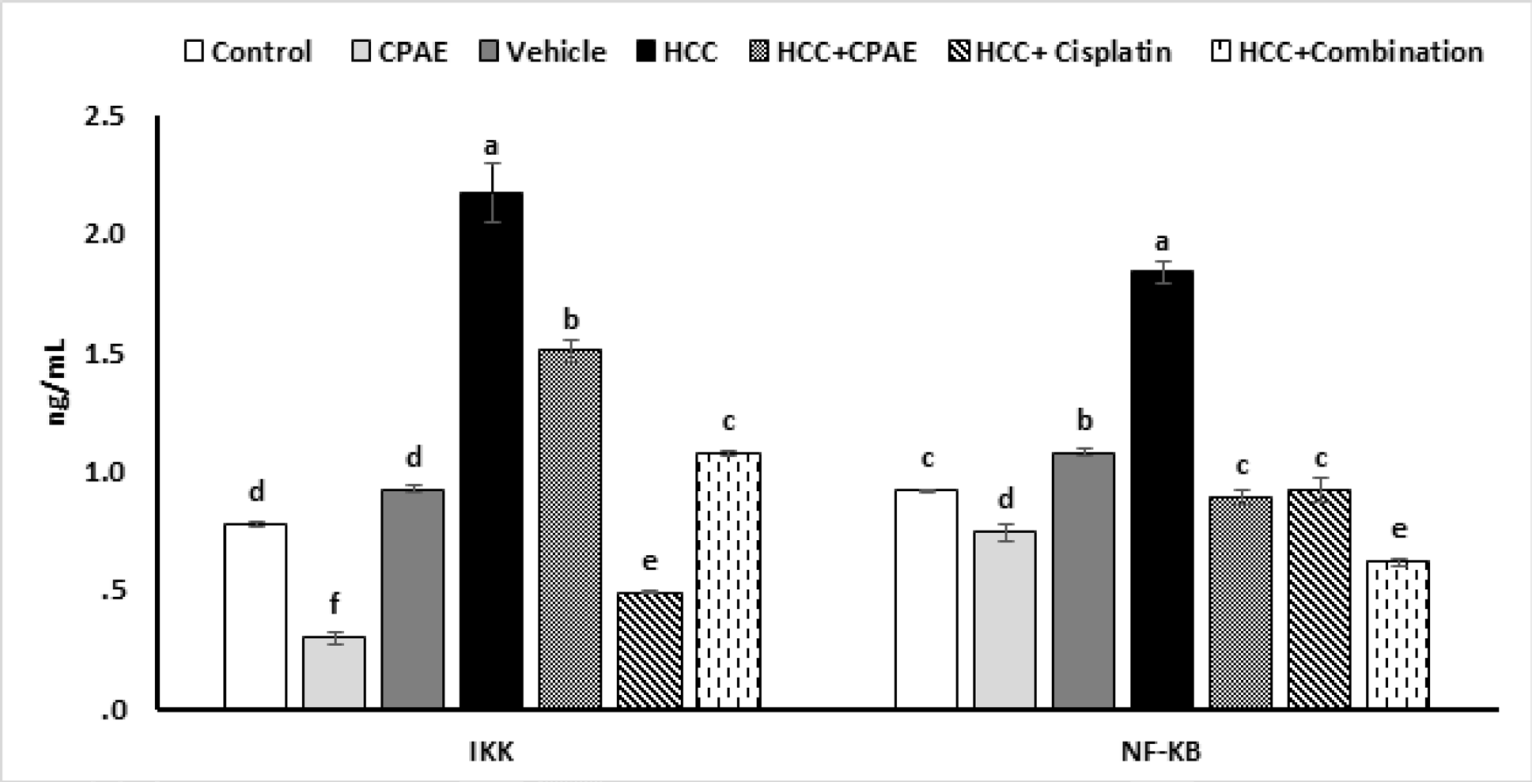
Hepatic protein of NF-KB and IKK in in different studied groups. Data were expressed as mean ± SD, *n* = 6. Mean values in the same column with common superscript letters (a, b, c, d, e, f) are not significant while mean values with different superscript letters are significantly different using the one-way ANOVA Test followed by the Tukey post hoc test at *p* > 0.05

**Fig. 5:**
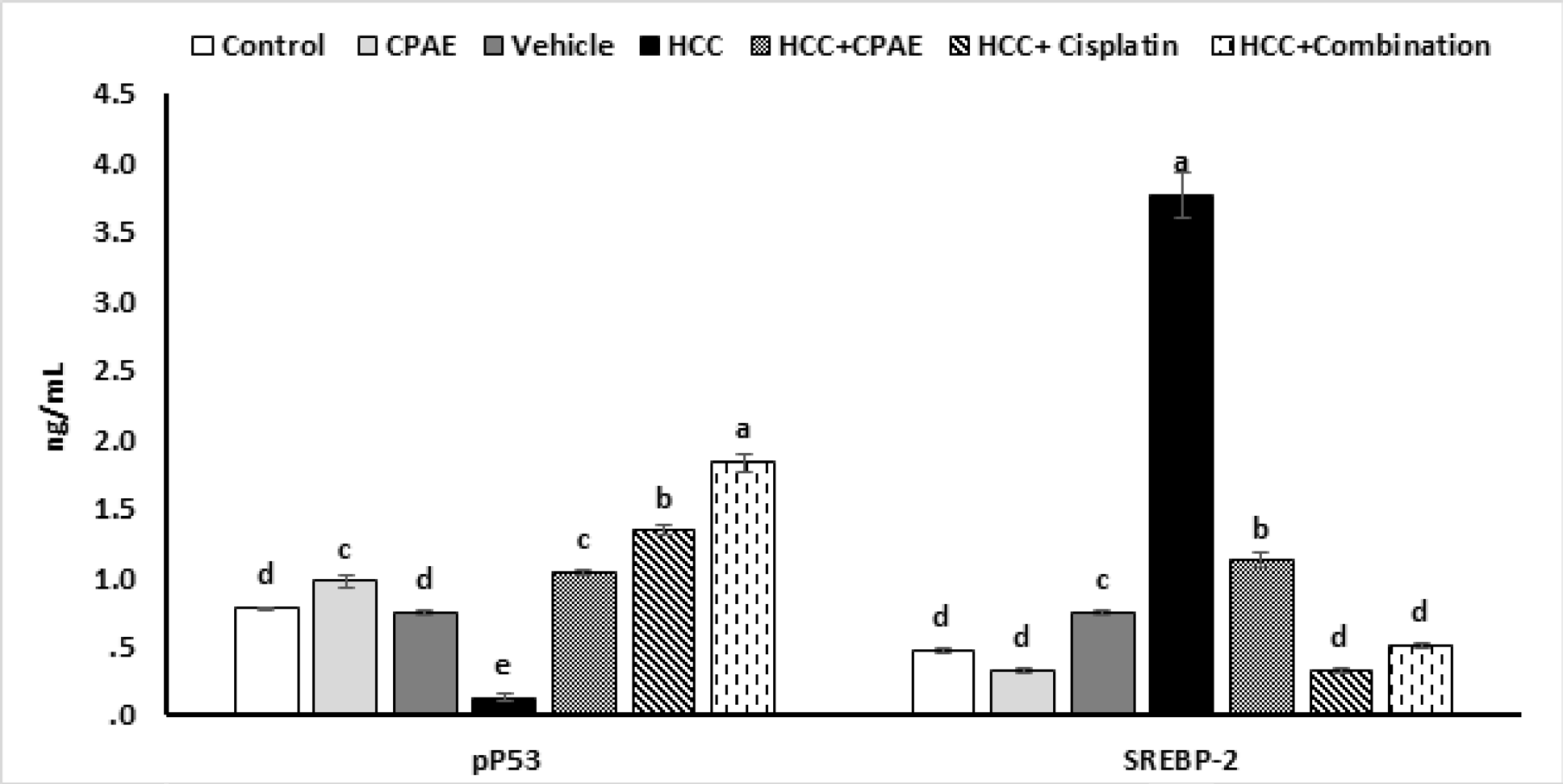
Hepatic protein of pP53 and SREBP-2 in different studied groups. Data were expressed as mean ± SD, *n* = 6. Mean values in the same column with common superscript letters (a, b, c, d, e) are not significant while mean values with different superscript letters are significantly different using the one-way ANOVA Test followed by the Tukey post hoc test at *p* > 0.05

**Fig. 6:**
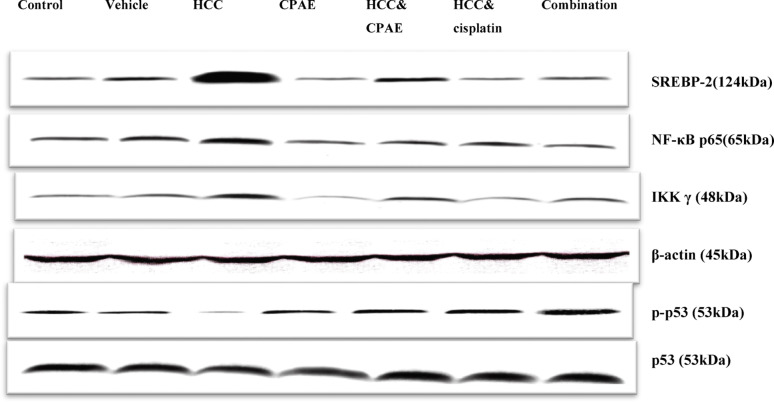
Western blot bands of SREBP-2, NF-κB p65, IKK γ, β-actin, p-p53, p53 in in different studied groups

In Figure (4, 5 and 6), we find the hepatic proteins NF-κB p65, IκK γ, pP53, and SREBP2. The healthy rats supplemented with CPAE showed a significant decrease in the hepatic proteins NF-κB and IκK compared to control rats. On the other hand, pP53 showed significant elevation compared to the control group. While the vehicle group showed significant elevations of NF-κB and IκK compared to CPAE and control rats, pP53 conversely showed a significant decrease compared to CPAE and didn’t show any effect compared to control. The CPAE and vehicle rats didn’t show any significant effect on SREBP-2 protein compared to control rats. On the other hand, the HCC rats showed significant up-regulation of the hepatic proteins (NF-κB, IκK, and SREBP-2) compared to the control, CPAE, and vehicle groups. In contrast, the pP53 protein showed significant downregulation compared to control, CPAE, and vehicle rats. However, the treatment of HCC rats with CPAE, cisplatin, or their combination significantly corrected all the hepatic protein derangement. In most of the studied parameters, the most favorable outcome was observed in the HCC rats receiving combination therapy, which exerted a synergistic effect on all hepatic protein parameters. These findings were confirmed by the calculated CI values which less than (Table 5).

**Table 6:** Analysis of CPAE/Cisplatin combination

Parameters	CI	Effect
SOD (U/mg. Protein)	0.62 ± 0.05	Synergistic
GST (U/mg. Protein)	0.52 ± 0.03	Synergistic
GSH (mg/mg. Protein)	0.63 ± 0.06	Synergistic
NO (nmol/mg. Protein)	0.54 ± 0.05	Synergistic
MDA (nmol/mg. Protein)	0.49 ± 0.05	Synergistic
XO (U/mg. Protein)	0.47 ± 0.02	Synergistic
GPx (U/mg. Protein)	0.62 ± 0.11	Synergistic
ATPase (nmol/mg. Protein)	0.53 ± 0.1	Synergistic
Cholesterol (mg/dL)	0.58 ± 0.07	Synergistic
Triglyceride (mg/dL)	0.48 ± 0.04	Synergistic
FBS (mg/dL)	0.52 ± 0.04	Synergistic
Creatinine (mg/dL)	0.51 ± 0.03	Synergistic
B.Urea (mg/dL)	0.47 ± 0.06	Synergistic
ALT (U/L)	0.53 ± 0.08	Synergistic
AST (U/L)	0.49 ± 0.05	Synergistic
ALP(U/L)	0.45 ± 0.04	Synergistic
Bilirubin (mg/dL)	0.69 ± 0.24	Synergistic
T.Protein (g/dL)	0.51 ± 0.02	Synergistic
Albumin (g/dL)	0.52 ± 0.02	Synergistic
AFP (ng/mL)	0.41 ± 0.03	Synergistic
AMPK (Fold change)	0.78 ± 0.06	Synergistic
PGC1α (Fold change)	0.7 ± 0.06	Synergistic
TFAM (Fold change)	1.25 ± 0.06	Reverse antagonist
SIRT1 (Fold change)	0.61 ± 0.1	Synergistic
iNOS (Fold change)	0.46 ± 0.03	Synergistic
IKK (ng/mL)	0.59 ± 0.02	Synergistic
NF-kB (ng/mL)	0.37 ± 0.01	Synergistic
pP53(ng/mL)	0.75 ± 0.03	Synergistic
SREBP-2 (ng/mL)	0.39 ± 0.01	Synergistic

Combination index value of = 1, ˂1, ˃1, and indicate additive, synergistic, and antagonism, respectively. Value are means ± SD.

### Impact of CPAE and/or cisplatin on liver histology

#### Control, CPAE, and vehicle groups

**Fig. 7:**
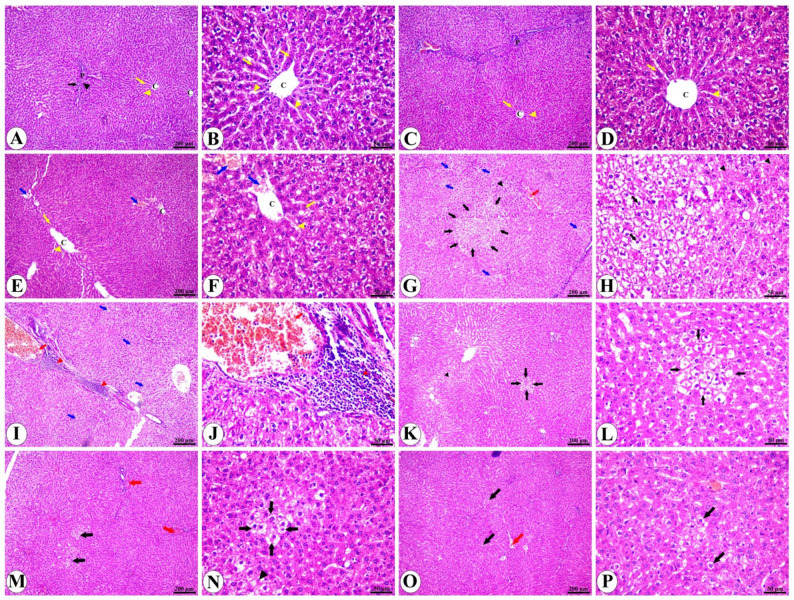
photomicrograph of H&E stained hepatic parenchyma of control (**A**, **B**), carob (**C**, **D**) and vehicle (**E**, **F**) groups showing normal hepatic architecture with central vein (C), polyhedral shaped hepatocytes arranged in a cord like pattern (yellow arrow) separated by hepatic sinusoids (yellow arrow head), (P) containing hepatic artery (black arrow head), bile duct (black arrow) in addition to mild congestion of some hepatic blood vessels in vehicle group (blue arrows). HCC group (**G**, **H**, **I** and **J**) showing loss of hepatic architecture with presence of HCC nodules (black arrows), small aggregations of clear cells (blue arrows), congestion of blood vessels (red arrows), area of hepatocellular degeneration (black arrow head) and massive infiltration of mono nuclear cells in the portal area (red arrow heads). HCC-Cisplatin treated group (**K**, **L**) showing normal hepatic architecture with presence small nodules of HCC (black arrows), small areas of hepatocellular degeneration (black arrow heads). HCC-CPAE treated group (**M**, **N**) showing normal hepatic architecture with presence small nodules of HCC (black arrows), small areas of hepatocellular degeneration (black arrow heads) and congestion of blood vessels (red arrows). Cisplatin and CPAE treated group (**O**, **P**) showing significant amelioration of hepatic tissue with presence small aggregations of HCC (black arrows), and mild congestion of blood vessels (red arrows).

Liver sections revealed normal hepatic architecture with a central vein (C) in the center of the hepatic lobule surrounded by polyhedral-shaped hepatocytes. These hepatocytes, with centrally located nuclei arranged in a cord-like pattern (yellow arrow) and separated by hepatic sinusoids (yellow arrowhead). The portal area (P) containing the hepatic artery (black arrowhead) and bile duct. The vehicle group showed minor congestion of some hepatic blood vessels (blue arrows) (Fig. 7A-C).

#### HCC control group

Hepatic parenchyma exhibited extensive toxic damage induced DEN/CCl_4_, characterized by marked disruption of normal hepatic architecture. Key features included the presence of HCC nodules (black arrows) composed of clear cells with pale cytoplasm and hyperchromatic nuclei present in a small aggregation (blue arrows), congested blood vessels (red arrows), and areas of hepatocellular degeneration (black arrowhead). Affected hepatocytes contained large fat-filled vacuoles in their cytoplasm, mild pyknotic nuclei, and congested, dilated blood vessels with scant collagen fibers (Fig. 7G- J).

### Cisplatin-HCC treated group

Liver architecture was largely restored, though there were small nodules of HCC (black arrows). Other findings included small areas of hepatocellular degeneration (black arrowheads) and necrotic foci with inflammatory cell infiltration. Additional features included cytoplasmic vacuolation, dissolution of hepatic cord, and mild biliary proliferation. Moderate numbers of apoptotic and degenerative hepatocytes are also preserved (Fig. 7K-L).

#### CPAE-treated HCC group

Hepatic sections showed a normal central vein. The hepatocytes were arranged in a cord with the presence of small nodules of HCC (black arrows) and congestion of blood vessels (red arrows). The proliferation of oval cells in some sections may indicate the distribution of necrotic cells. Hepatocytes contained large fat-filled vacuoles and mild pyknotic nuclei. The blood vessels were congested and dilated with a few strands of collagen fibers (Figures M, N).

#### Combination therapy (CPAE + Cisplatin)-HCC treated group

Combination therapy markedly restored hepatic architecture, although occasional hepatocytes displayed karyomegaly and small HCC foci (black arrows) and necrotic nuclei. The tissue also exhibited collagen fiber depositions in the portal triad and mildly congested blood sinusoids (red arrows) (Figures O, P).

Table 6 shows that the HCC rats had significant increase in necrotic and inflammatory cells, accompanied by severe hemorrhage, deposition of fats, and marked dilation of blood vessels compared to the control groups. Treatment of HCC rats with CPAE, cisplatin, or their combination partially ameliorated all abnormal histological changes, for most of the studied parameters, the best effects were observed in the HCC rats treated with combination therapy.

**Table 7:** The statistical variation of the liver histological parameters in different studied groups

Item	Necrotic cells	Inflammatory cells	Hemorrhage	Fatty change	Blood vessels
Control	0.6^e^ ± 0.2	0.6^e^ ± 0.2			Average
CPAE	0.4^e^ ± 0.2	1.2^e^ ± 0.4	−		Average
Vehicle	1.1^e^ ± 0.4	1.4^e^ ± 0.5	+		Average
HCC	84.4^a^ ± 1.3	52.4^a^ ± 0.9	+++	+++	Markedly dilated
HCC+CPAE	57.6^b^ ± 2.3	27^c^ ± 1.3	+	+	Dilated
HCC+cisplatin	66.6^c^ ± 1.2	41.2^b^ ± 0.6	++	++	Moderate dilated
HCC+Combination	41.4^d^ ± 3.8	24.2^d^ ± 0.6	+	+	Average

Data were expressed as mean ± SD, *n* = 6. Mean values in the same column with common superscript letters (a, b,c, d,e) are not significant while mean values with different superscript letters are significantly different using the one-way ANOVA Test followed by the Tukey post hoc test at *p* > 0.05 (Table 7).

## Discussion

In this study, an HCC model was established in a two-step process through the introduction of two different types of carcinogenic compounds, DEN/CCl_4_. DEN is the most widely used liver carcinogenic substance that causes the generation of reactive oxygen species (ROS), leading to oxidative stress and DNA damage in an irreversible process (You et al. 2021). Second, CCl_4_ has been used as a tumor promoter by damaging cellular structures, leading to liver inflammation, fibrosis, cirrhosis, and ultimately HCC development, which mimics what occurs in human HCC (Santos et al. 2017). These effects of DEN/CCl_4_ resulted in histological changes toward the HCC phenotype, abnormalities in hepatic serum parameters, and AFP levels that were markedly elevated in HCC-rats compared to the control rats as an indicator of hepatocarcinogenicity. These results have been frequently observed elsewhere (Abdel-Hamid et al. 2021).

Histologically, DEN/CCl4 displayed severe hepatocellular disorganization, necrosis, and vascular dilatation, these pathological features are consistent with previous models of chemically induced HCC (Hamza et al. 2021, Abass et al. 2018, Jain 2014). These alterations were accompanied by elevated liver enzymes and AFP, confirming successful tumor induction.

The induction of HCC in rats using DEN/CCl4 is associated with metabolic abnormalities, as evidenced by the significantly increased serum fasting blood sugar and triglyceride levels. This elevation may reflect the impairment of liver function, mainly in carbohydrate and lipid metabolism (Bashandy et al. 2023, Arboatti et al. 2018, Llovet et al. 2021). Our finding suggests the mechanistic role of mitochondrial dysfunction and the disturbed AMPK signaling pathway. AMPK-mediated metabolic reprogramming, a well-known hallmark of cancer, provides a key link between hepatocarcinogenesis and the observed systemic energy imbalance, driving abnormal gluconeogenesis and accumulation of lipids.

These metabolic abnormalities in HCC-rats were associated with dysregulation of the hepatic expression of genes controlling hepatic metabolism and mitochondrial biogenesis, including AMPK, PGC-1α, TFAM, SIRT1, and SREBP-2. The observed suppression of AMPK, PGC-1α, and TFAM suggests impaired mitochondrial biogenesis in DEN/CCl_4_-induced HCC. This aligns with reports that AMPK downregulation promotes carcinogenic transformation by reducing mitochondrial turnover and oxidative metabolism [Rabinovitch et al. (2017), Wu et al.(2024), Mastropasqua et al. (2018), Elazab et al. (2025)]. The concomitant decrease in PGC-1α and TFAM further supports mitochondrial dysfunction as a central event in hepatocarcinogenesis (Zhao et al. 2021, Kang et al. 2018, Silva Ramos et al. 2019).

Consistent with the observed mitochondrial dysfunction, the hepatic tissues of HCC rats exhibited a state of oxidative stress, as demonstrated by elevated levels of the lipid peroxidation end product malondialdehyde (MDA). This oxidative stress results from both the induced production of ROS and RNS, accompanied by impaired of antioxidant defense systems, including inhibition of SOD, GPx, and GST activities and marked depletion of GSH. We propose that the initial mitochondrial impairments (downregulated PGC-1α/TFAM) serve as the primary source of increased ROS. This excess ROS subsequently overwhelm and deplete antioxidant defense system. This establishes a vicious cycle where oxidative stress causes mitochondrial damage, eventually promoting the progression of HCC, as supported by previous studies (Abdel-Hamid et al. 2021, Hsiao et al. 2024, Ahmad et al. 2025, Gani et al. 2019).

At the inflammatory level, HCC rats showed significant elevations in the inflammatory markers NF-κB and IκK compared to control rats, which reflects the extent of inflammation and the initiation of fibrotic reactions. Their higher levels indicate poor prognosis in liver pathologies and predispose them to the development of cancer; the obtained results have been documented in other reports (Hamza et al. 2021, El-Magd et al. 2019, Li et al. 2007).

AMPK is known to suppress inflammation by reducing pro-inflammatory markers and NF-κB levels (Xiang et al. 2025). We observed an upregulation of SIRT1 in our HCC model. The role of SIRT1 in hepatocarcinogenesis is context-dependent often acting as a tumor suppressor in early stages but exhibits oncogenic action in established disease. Within our model, characterized by suppression of AMPK and constitutive NF-κB activation, the upregulation of SIRT1 is likely maladaptive. This interpretation is supported by studies demonstrating that SIRT1 can promote tumor progression by enhancing the NF-κB signaling pathway and facilitating cancer cell survival and metastasis (Fu et al. 2024, Zheng et al. 2020, Li et al. 2016). Therefore, we propose in this setting, that SIRT1 upregulation is not a protective mechanism but a contributor to tumorigenesis. The induced NF-κB pathway may interpret the enhanced expression of iNOS in the hepatic tissues of HCC rats, resulted in the production of a large amount of NO, as documented in the present study, leading to inflammation and even tumor initiation and progression (Zeng et al. 2025).

The adjuvant use of natural products with cisplatin represents a promising approach to enhance therapeutic efficacy and mitigate toxicity in HCC. This concept is supported by agent such as, metformin, which augment the chemosensitivity of hepatocarcinoma cells to cisplatin through AMPK pathway activation (Dong et al. 2017). Also, Kanglaite which potentiates cisplatin’s antitumor effect by inhibiting CKLF1-mediated NF-κB signaling (Chen et al. 2021). Our finding position CPAE within this emerging category of chemosensitizing adjuvant. CPAE appear to act via multimodal mechanism, concurrently targeting the AMPK/PGC-1α axis to restore mitochondrial biogenesis, suppressing NF-κB to attenuate inflammation, and enhancing endogenous antioxidant defenses. This coordinating action likely underpins its dual benefit of both sensitizing tumors to cisplatin and directly counteracting its dose limiting nephrotoxicity.

Treatment of HCC rats with cisplatin significantly ameliorated the HCC conditions at different levels: histological, biochemical, metabolic, inflammatory, and redox. However, cisplatin-induced nephrotoxicity is indicated by the elevation of blood urea levels. Co-treatment of cisplatin-treated HCC rats with CPAE significantly boosted the antitumor effects of cisplatin at all levels and significantly prevented its nephrotoxicity. The nephroprotective effect of CPAE observed in our study is consistent with previous findings on carob, which exhibited efficacy against doxorubicin-induced nephrotoxicity (Ishteyaque 2024). This aligns with many evidences for natural antioxidant including ginseng, N-acetylcysteine and vitamin E, all of which have shown promise in mitigating drug-induced kidney injury (Atta et al. 2023, Huang et al. 2019, Zavala-Valencia et al. 2024, Fang et al. 2021).

Cisplatin treatment showed mild amelioration of the histological abnormalities in HCC rats; however, CPAE-adjuvant treatment with cisplatin showed marked improvements in the hepatic histology and was able to reverse hepatocytic damage and integrity of cells and protect liver cells from inflammation, necrosis, fibrosis, and steatosis, thereby suggesting the restoration of liver architecture in HCC rats. Unexpectedly, the treatment of HCC rats with CPAE alone markedly ameliorates the histological changes in HCC, similar to or even better than the effects of cisplatin. Moreover, similar patterns of variations were noted in the serum parameters: AFP, ALT, AST, ALP, bilirubin, albumin, urea, and creatinine, with the best ameliorative effects observed in the HCC rats co-treated with cisplatin and CPAE, followed by those treated with CPAE alone and those treated with cisplatin alone.

At the metabolic level, CPAE has potential glucose-lowering, lipotropic and metabolic-boosting effects, improving hepatic metabolic performance and enhancing mitochondrial biogenesis and function. This observation was supported by the study by Tanaka et al. (2020), who revealed that gallic acid suppresses hepatic lipid accumulation and inflammation (Tanaka et al. 2020). The lipid-lowering action of CPAE may be mediated through the inhibition of hepatic cholesterol biosynthesis, as indicated by the suppression of SREBP-2 and/or reduction of lipid absorption in the intestine. Since glucose is greatly consumed by cancer cells during metabolism to fuel malignant proliferation, targeting increased glycolysis could offer therapeutic benefits in cancer (Atya et al. 2021).

In the present study, HCC induction led to a significant downregulation of AMPK and PGC-1α, consistent with a state of mitochondrial dysfunction and metabolic reprogramming favorable for cancer growth. Treatment with CPAE, both alone and in combination with cisplatin, markedly reversed this suppression, upregulating the AMPK-PGC-1α-TFAM signaling cascade.

The metabolic effects of CPAE in HCC rats were associated with significant upregulation of the expression of AMPK, PGC-1α, and TFAM compared to untreated HCC rats or even control rats, whereas treatment with cisplatin alone resulted in mild effects on these parameters. In addition, CPAE treatment significantly downregulated SREBP-2 and SIRT1 compared to those in untreated HCC rats. While cisplatin itself can activate AMPK, activation of AMPK by CPAE in the liver was very successful not only in reinforcing cisplatin sensitivity but also in improving the expression of most cancer markers analyzed and protecting normal tissues against the toxic effects of cisplatin.

At the inflammatory level, cisplatin and/or CPAE treatments significantly downregulated NF-κB, IκK, and iNOS compared to untreated HCC rats, indicating their potential anti-inflammatory action. We assume that the downregulation effect of CPAE on iNOS may be due to CPAE significantly inhibiting NO production, as observed in our in-vitro study, which also attenuated RNS production in the liver of HCC rats and decreased inflammation in HCC, as described by the significant reduction in inflammatory cells, necrotic cells, hemorrhage, and inhibition of NF-κB.

Regarding the redox status, the treatment of HCC rats with CPAE alone or in combination with cisplatin significantly boosted the activities of the antioxidant enzymes SOD, GPx and GST, and the concentration of GSH in the hepatic tissues and significantly decreased the elevated levels of NOx, XO, and MDA compared to the untreated HCC rats. These results are in agreement with Martić Nicola et al., who found that carob pulp flower extract had a hepatoprotective impact against paracetamol-induced hepatic and kidney oxidative stress (Martić et al. 2022). The ameliorative effects of CPAE on antioxidant parameters have been previously described (El-haskoury et al. 2018, Al-Olayan et al. 2016). The observed antioxidant improvement could be attributed to reduced DNA carcinogen interactions, suppression of lipid peroxidation and protein oxidation, and inhibition of ROS formation consistent with radical scavenging activity (Aly et al. 2019). We propose that the underline chemotherapeutic activity is not attributed to a single component, but rather than the synergistic impact of its major phenolic content; gallic acid, catechin, and protocatechuic acid (PCA). Furthermore, these results were supported by our previous study, in which HPLC analysis showed the presence of these compounds in CPAE, and our results align with documented mechanisms for each. Gallic acid ameliorates hepatic liver enzymes, tissue architecture and suppresses HCC tumorigenesis in vitro and in vivo by targeting the MALAT1-Wnt/β-catenin signaling axis (Aglan et al. 2017, Shi et al. 2021). PCA ameliorate HCC through restoring liver function and hepatocyte morphology, boosting antioxidant activity, inhibiting cancer cell metastasis via downregulating the Ras/Akt/NF-κB pathway (Gani et al. 2019, Lin et al. 2011). Moreover, catechins possess potent antioxidant, anti-inflammatory, anticancer, and chemopreventive properties against a variety of tumors, including HCC (Shimizu et al. 2015). Collectively, these findings demonstrate that the treatment with CPAE protects hepatocytes from damage and dysfunction caused by DEN/CCl_4_-induced hepatocarcinogenesis. Furthermore, the combination of CPAE with cisplatin exerts synergistic chemotherapeutic effect by restoring redox homeostasis, suppressing inflammation, and ameliorating mitochondrial dysfunction in HCC-bearing rats.

### Limitations of the study

While the present study provides compelling evidence that CPAE exert ant-HCC activity both alone and as adjuvant with cisplatin. However, there is several limitations should be known. The chemically induced animal models used in the study restricts direct exploration ton human HCC because of interspecies difference in drug metabolism, immune response, and tumor microenvironment. Second, small sample size reducing statical power. Absence of pharmacokinetic and pharmacodynamic data on CPAE constituent, which restrain optimization of dosing regimens and prediction of effective human doses. Also, the molecular mechanism underling synergism interaction between CPAE and cisplatin remain incompletely elucidated. The relative short treatment duration (4weeks) may not reflect long term efficacy, toxicity across multiple organs. Moreover, the use of male animal models precludes the assessment of any sex-specific variation in therapeutic response.

## Conclusions

This study demonstrates that carob pod aqueous extract (CPAE) confers substantial hepatoprotective and chemopreventive effects against chemically induced hepatocellular carcinoma in rats, both as a monotherapy and in combination with cisplatin. CPAE ameliorates liver dysfunction, metabolic disturbances, oxidative stress, and inflammatory signaling while restoring mitochondrial biogenesis and normalizing gene/protein expression profiles associated with tumor suppression. Notably, the combination of CPAE with cisplatin not only enhances anticancer efficacy but also alleviates cisplatin-induced nephrotoxicity, offering a dual benefit. These findings highlight the potential of CPAE as an adjunct to conventional chemotherapy, with implications for improving therapeutic outcomes and reducing side effects in HCC. Future research will focus on the clinical translation of these finding: the essential next steps include: First, establishing the pharmacokinetic and toxicological profiles of CPAE and its active components to determine an effective and safe human dosing regimen. Second, conducting mechanistic studies to investigate interactions between the bioactive compounds of CPAE and chemotherapeutic drugs will aid in optimizing combination therapy. Third, an early-phase clinical trial is necessary to evaluate the safety, initial effectiveness, and tolerability of CPAE, especially as an adjuvant to cisplatin in HCC patients. These trials might also explore the potential of CPAE to mitigate cisplatin-induced nephrotoxicity, thereby improving patient outcomes. Overall, these steps are crucial for translating preclinical promise into clinical application, introducing a novel natural adjuvant regimen for HCC therapy.

### Recommendations

Given the significant protective and synergistic antitumor effects observed with CPAE and cisplatin combination therapy, it is recommended that further preclinical studies, including pharmacokinetic and toxicological evaluations, be conducted to facilitate clinical translation. Investigations should extend to exploring CPAE’s efficacy in different HCC models and its interactions with other chemotherapeutic agents. Clinical trials are warranted to assess safety, optimal dosage, and therapeutic efficacy in human patients. Additionally, mechanistic studies at the cellular and molecular levels should be pursued to fully delineate the pathways involved in CPAE-mediated modulation of oxidative stress, mitochondrial function, and inflammatory responses. These efforts could pave the way for the development of novel integrative therapeutic protocols for HCC and potentially other malignancies.

## Data Availability

The datasets generated and analyzed during the current study are available from the corresponding author upon reasonable request.

## Declarations

### AI statement

AI-based tools were used solely for language editing and manuscript structuring to improve clarity and readability. All scientific data collection, analysis, and interpretation were performed personally by the authors, who take full responsibility for the content.

## Abbreviations

Abbreviation: Full term
HCC: Hepatocellular carcinoma
CPAE: Carob pod aqueous extract
DEN: Diethylnitrosamine
CCl₄: Carbon tetrachloride
AMPK: AMP-activated protein kinase
PGC-1α: Peroxisome proliferator-activated receptor-gamma coactivator 1α
TFAM: Mitochondrial transcription factor A
SIRT1: Sirtuin-1
iNOS: Inducible nitric oxide synthase
NF-κB: Nuclear factor kappa B
IκK: Inhibitor of kappa B kinase
p53: Tumor protein p53
pP53: Phosphorylated p53
SREBP-2: Sterol regulatory element-binding protein-2
ROS: Reactive oxygen species
NO: Nitric oxide
MDA: Malondialdehyde
XO: Xanthine oxidase
GSH: Glutathione
GPx: Glutathione peroxidase
GST: Glutathione-S-transferase
SOD: Superoxide dismutase
AFP: Alpha-fetoprotein
ALT: Alanine aminotransferase
AST: Aspartate aminotransferase
ALP: Alkaline phosphatase
TC: Total cholesterol
TG: Triglycerides
FBS: Fasting blood sugar
BW: Body weight
ELISA: Enzyme-linked immune-sorbent assay
RT-PCR: Reverse transcriptase-polymerase chain reaction
qRT-PCR: Quantitative real-time reverse transcriptase-polymerase chain reaction
SD: Standard deviation
